# GLYI4 Plays A Role in Methylglyoxal Detoxification and Jasmonate-Mediated Stress Responses in *Arabidopsis thaliana*

**DOI:** 10.3390/biom9100635

**Published:** 2019-10-22

**Authors:** Silvia Proietti, Gaia Salvatore Falconieri, Laura Bertini, Ivan Baccelli, Elena Paccosi, Antonio Belardo, Anna Maria Timperio, Carla Caruso

**Affiliations:** 1 Department of Ecological and Biological Sciences, University of Tuscia, 01100 Viterbo, Italy; 2 Institute for Sustainable Plant Protection, National Research Council of Italy, Sesto Fiorentino, 50019 Florence, Italy

**Keywords:** GLYI4, methylglyoxal, methyl-jasmonate, arabidopsis

## Abstract

Plant hormones play a central role in various physiological functions and in mediating defense responses against (a)biotic stresses. In response to primary metabolism alteration, plants can produce also small molecules such as methylglyoxal (MG), a cytotoxic aldehyde. MG is mostly detoxified by the combined actions of the enzymes glyoxalase I (GLYI) and glyoxalase II (GLYII) that make up the glyoxalase system. Recently, by a genome-wide association study performed in Arabidopsis, we identified GLYI4 as a novel player in the crosstalk between jasmonate (JA) and salicylic acid (SA) hormone pathways. Here, we investigated the impact of *GLYI4* knock-down on MG scavenging and on JA pathway. In *glyI4* mutant plants, we observed a general stress phenotype, characterized by compromised MG scavenging, accumulation of reactive oxygen species (ROS), stomatal closure, and reduced fitness. Accumulation of MG in *glyI4* plants led to lower efficiency of the JA pathway, as highlighted by the increased susceptibility of the plants to the pathogenic fungus *Plectospherella cucumerina*. Moreover, MG accumulation brought about a localization of GLYI4 to the plasma membrane, while MeJA stimulus induced a translocation of the protein into the cytoplasmic compartment. Collectively, the results are consistent with the hypothesis that GLYI4 is a hub in the MG and JA pathways.

## 1. Introduction

Plants have developed efficient detection mechanisms and effective signal transduction pathways to respond to diverse biotic and abiotic stresses and activate finely tuned regulatory mechanisms, largely orchestrated by small molecules [1]. Among them phytohormones such as salicylic acid (SA), jasmonic acid (JA), ethylene (ET), and abscisic acid (ABA), are considered as the main players in coordinating signaling pathways involved in the adaptive response of plants to its (a) biotic environment, together with cytokinin, brassinosteroids, and auxin [1,2,3,4]. Moreover, many defense strategies are regulated by more than one phytohormone and often multiple processes are induced simultaneously. Consequently, antagonistic and synergistic interactions between diverse hormone signal transduction pathways, the so-called cross-talk, add another layer of regulation [1]. 

Beside hormones, plants under stress can produce small molecules, and depending on their concentration they can be either toxic or can act as signal molecules. For instance, in stressful conditions and also in response to primary metabolism alterations can produce toxic aldehydes among which methylglyoxal (MG) appears as the most ubiquitous [5,6,7,8,9,10]. It is an alpha-oxoaldehyde compound, highly reactive and cytotoxic, produced as a side product of various metabolic reactions such as glycolysis, protein degradation, lipid peroxidation, and photosynthesis [11,12]. It has been extensively reported that MG at low doses plays a role as signal molecule in bacteria [13], yeast [14,15], animals [16,17,18,19], and plants [7,20,21]. In addition, in plants MG exerts a positive effect on shoot differentiation and morphogenesis [22] and a negative effect on seed germination and root elongation [6]. Moreover, the presence of MG has been also correlated to the opening and closing of stomata [23]. MG triggers stomatal closure by inducing reactive oxygen species (ROS) accumulation in the guard cells through the Ca^2+^-dependent pathway. Unfortunately, at higher concentration, MG is detrimental for the cell as it reacts with major macromolecules including DNA, RNA, and proteins and lipids modifying or disrupting their physiological functions [10,11,24]. MG-affected genes are also involved in hormone signaling, cell-to-cell communications, and chromatin remodeling [10]. The only way to counteract the toxic effects of MG is its removal by an efficient detoxification system [11]. 

Glyoxalase (GLY) system is the major MG detoxifying pathway in all organisms including bacteria, yeast, humans, plants, and animals [11]. The GLY system is a two-step scavenging pathway involving two phylogenetically distinct enzymes, glyoxalase I (GLYI) and glyoxalase II (GLYII) able to detoxify MG [25]. In the first step, MG is scavenged by reduced glutathione (GSH) forming a hemithioacetal, a non-enzymatic adduct, which is the real substrate for the first reaction catalyzed by GLYI, leading to S-D-lactoylglutathione. In a second step, S-D-lactoylglutathione is converted into D-lactate by the GLYII, thus releasing GSH [25]. Another enzyme, D-lactate dehydrogenase (D-LDH) is linked with MG detoxification since it catalyzes the oxidation of the end product of the glyoxalase system, D-lactate, into D-pyruvate which eventually enters into TCA cycle for energy production [14]. GLYI overexpressing cells were highly efficient in detoxifying MG showing also better growth than wild-type cells [11]. In addition, a shorter route for MG detoxification mediated by glyoxalase III (GLYIII) has been proposed in few organisms [23]. In particular, GLYIII transforms MG into D-lactate in a single step, without using glutathione. Interestingly, its activity appeared significantly lower than the GLYI/GLYII system [23]. 

In plants, glyoxalases exist as a multigene family whose functions probably go beyond the canonical role of MG detoxification [23]. Indeed, increase in glyoxalase enzyme activities occurs in plants in response to osmotic stress, extreme temperatures, heavy metals, and exposure to stress-related hormones, including methyl jasmonate (MeJA), ABA, and SA [10,26,27]. Accordingly, several biotic/abiotic stress-responsive *cis-acting* elements have been found in the promoter regions of *GmGLYI* and *GmGLYII* family members from *Glycine max*, suggesting an hormone-regulated gene expression [28]. Moreover, transgenic plants over-expressing either *GLYI* or *GLYII* showed improved tolerance against several abiotic stresses, as drought, salinity, and heavy metal in various plant species by maintaining the basal level of MG and mitigating oxidative stress [29,30,31,32,33,34,35]. Based on these observation, MG and glyoxalases could be targeted as potential biomarkers for plant stress physiology [21].

The Arabidopsis genome annotation includes 11 *GLYI* genes, encoding for 22 proteins that are members of the vicinal oxygen chelate (VOC) superfamily [36,37]. The molecular mechanism, functional roles, and subcellular localization of the different isoforms are still to be unraveled. Among GLYI members, GLYI4 is highly expressed in pistil tissue, stigma, and dry pollen, suggesting a role in development and reproduction [23]. Moreover, *GLYI4* transcripts are highly abundant in roots, flowers, and seeds [25,37]. In Arabidopsis *AtGLYI4* is likely a key component of the gene network interaction responsible for detoxifying MG [37], although it has not yet been demonstrated experimentally. Expression of *AtGLYI4* is also induced in response to abiotic stresses in either shoot and root tissues [37]. Recently, by a genome-wide association study we identified GLYI4 as a novel player in the SA-JA crosstalk [38]. This novel role has been confirmed by T-DNA insertion mutant analysis. In particular, the *glyI4* knock-down mutant was insensitive to SA-mediated suppression of MeJA-induced *PDF1.2* gene expression, while it displayed wild-type levels of SA-induced *PR-1* gene expression. Moreover, the lack of SA-mediated antagonism on the JA pathway in *glyI4* mutant was associated with enhanced level of resistance against the fungal pathogen *B. cinerea* [38]. 

In this study, we first investigated the impact of GLYI4 on MG scavenging and on plant health. In the T-DNA insertion line *glyI4* we observed compromised MG scavenging, ROS accumulation, and stomatal closure. All these phenomena could be responsible for the altered fitness parameters observed in *glyI4* plants. In addition, we investigated the impact of MG on the JA pathway. Accumulation of MG in the mutant *glyI4* led to lower efficiency of the JA pathway, causing an increased susceptibility to the fungal necrotrophic pathogen *Plectospherella cucumerina*. Moreover, MG accumulation led to a subcellular localization of GLYI4 on the plasma membrane, while MeJA treatment induced a translocation of the protein into the cytoplasm. In-depth studies of MG interactions with plant hormones and the glyoxalase system will likely reveal more regulatory roles of MG in plant stress responses and tolerance. 

## 2. Materials and Methods

### 2.1. Plant Material and Growth Conditions

*Arabidopsis thaliana* T-DNA lines in Col-8 background (*glyI4*) were obtained from NASC (http://arabidopsis.info/) and genotyped as previously described [38]. Seeds of the Arabidopsis *glyI4* and Col-8 were sown in cultivation containers filled with autoclaved river sand. Sand was supplied with half-strength Hoagland solution [39]. (Sigma, Steinheim, Germany). To achieve a high relative humidity for germination, cultivation containers were enclosed in a tray with water and covered with a transparent lid. Seeds were stratified for two days at 4 °C in the dark to ensure a homogeneous germination after which the tray was moved to a growth chamber with an 8-h day/16-h night period, a temperature of 21 °C, and a light intensity of 100 µmol m^−2^ s^−1^. After eight days, the lids of the trays were slightly opened and gradually removed over a two-day period. Ten-day-old seedlings were transplanted to individual pots containing an autoclaved mixture of river sand and potting soil (1:1 (*v*:*v*)). Plants were watered from the bottom three times per week. From three weeks and onwards, the plants were supplied once a week with half-strength Hoagland solution.

### 2.2. Chemical Treatments

Five-week-old Arabidopsis plants (Col-8 and *glyI4* T-DNA line) were treated with MeJA (Serva, Brunschwig Chemie, Amsterdam, the Netherlands), MG (Sigma, Steinheim, Germany), or a combination of MeJA/MG, by dipping plants for about 5 s in a solution containing 100 μM MeJA or 10 mM MG or a combination of 100 μM MeJA/10 mM MG, all with 0.015% (*v*/*v*) Silwet L77 (Van Meeuwen Chemicals BV, Weesp, the Netherlands). MeJA was diluted from a 1000-fold concentrated stock in 96% ethanol. The mock solution contained 0.015% Silwet L77 only. Twenty-four hours after treatment, two leaves from each three plants per treatment were harvested. The harvested leaves were the fifth and 11th, both middle-aged. immediately frozen in liquid nitrogen and then stored at −80 °C until further analysis. 

### 2.3. RNA Extraction and RT-qPCR

Total RNA was isolated as previously described [40]. DNAse treatment was performed by using DNAse I (Fermentas, St. Leon-Rot, Germany) at a concentration of 0.5 U/g RNA. RevertAid H minus Reverse Transcriptase (Fermentas, St. Leon-Rot, Germany) was used to convert DNA-free total RNA into cDNA. PCR reactions were performed in optical 96-well plates (Applied Biosystems, Carlsbad, CA, USA) with an ABI PRISM^®^ 7900 HT sequence detection system using SYBR^®^ Green to monitor the synthesis of double-stranded DNA. A standard thermal profile was used: 50 °C for 2 min, 95 °C for 10 min, 40 cycles of 95 °C for 15 s and 60 °C for 1 min. Amplicon dissociation curves were recorded after cycle 40 by heating from 60 to 95 °C with a ramp speed of 1.0 °C min^−1^. Transcript levels were calculated relative to the Arabidopsis reference gene PP2AA3 [41] using the 2^−ΔΔCT^ method described previously [42]. Gene expression fold change was calculated relative to mock-treated wild-type plants. The Arabidopsis gene identifier (AGI) numbers of the studied genes are At5g44420 (*PDF1.2*), At1g15380 (*GLYI4*)*,* At1g13320 (*PP2AA3*). Primers are the following: *PDF1.2* (Fw: CACCCTTATCTTCGCTGCTCTT; Rv: GCCGGTGCGTCGAAAG), *GLYI4* (Fw: GAAGGAAGACGCAGGAAACC; Rv: TCGGCACAAGACAGAGACAT); *PP2AA3* (Fw: TAACGTGGCCAAAATGATGC; Rv: GTTCTCCACAACCGCTTGGT)

### 2.4. Total Protein Extraction and Western Blotting

Five-week-old Arabidopsis ecotype Col-8 and *glyI4* leaves (500 mg) were ground to fine powder in a pre-chilled mortar in the presence of liquid nitrogen. Extraction buffer, containing 50 mM HEPES pH 7.5, 1% (*w*/*v*) polyvinylpolypyrrolidone, 10 mM MgCl_2_, 1 mM EDTA, Triton 0.1%, and a cocktail of protease inhibitors (Roche, Basel, Switzerland), was added to the powder. The total extract was then centrifuged at 13,000× *g* for 15 min at 4 °C, and the clear supernatant was used for SDS-PAGE.

Western blot analysis was carried out following the method previously described [43]. Forty g of protein extract was separated by 15% (*w*/*v*) SDS-PAGE and electrophoretically transferred onto nitrocellulose membrane. Protein markers were used to assess molecular weights (SMOBIO-PM 2610, SMOBIO Technology, Hsinchu City, Taiwan). Following electroblotting, the membrane was blocked for 30 min in PBS buffer (6.5 mM Na_2_HPO4, 1.5 mM KH_2_PO4, 3 mM KCl, 0.15 M NaCl, pH 7.4) containing 0.5% (*v*/*v*) Triton X-100 and 3% (w/v) BSA and then incubated overnight at room temperature with a Mouse Anti-Methylglyoxal monoclonal antibody (dilution 1:1000) as primary antibody (STA-011, Cell Biolabs, San Diego, CA, USA). The immunoreactive bands were detected with HRP-conjugated goat-anti-mouse IgG (dilution 1:7000) as secondary antibody using the peroxidase substrate 4-chloro-1-naphthole (Sigma Aldrich, St. Louis, MO, USA). The same membrane was re-probed with Ponceau S (Advansta, Menlo Park, CA, USA) to normalize the protein loading, as indicated. Densitometric analysis of the selected MG protein-adduct band has been performed using Image J software (http://rsbweb.nih.gov/ij/).

### 2.5. Metabolite Extraction and LC-MS Analysis

Two hundred mg of Col-8 or *glyI4* leaves were finely ground in liquid nitrogen and powder was used for metabolite extractions. Briefly, cells were lysed by thermal shock (freezing/heating). A cold (−20 °C) solution of 60% methanol/40% chloroform was added to each tube. The tubes were mixed for 30 min and subsequently centrifuged at 1000× *g* for 1 min at 4 °C, before being transferred to −20 °C for 2–8 h. After thawing, liquid phases were recovered and samples were incubated at 4 °C for 20 min, centrifuged at 13,500× *g* for 10 min at 4 °C and the collected supernatants were dried to obtain visible pellets. Finally, the dried samples were re-suspended in water, 5% formic acid and transferred to glass autosampler vials for LC/MS analysis. Twenty l of samples (3 technical replicates) were injected into an Ultra High-Performance Liquid Cromatography (UHPLC) system (Ultimate 3000, Thermo Fisher Scientific, Waltham, MA, USA) and run in positive ion mode. A Reprosil C18 column (2.0 mm by 150 mm, 2.5 μm- Dr Maisch HPLC GmbH, Ammerbuch, Germany) was used for metabolite separation. Chromatographic separations were achieved at a column temperature of 30 °C and flow rate of 0.2 mL/min. A 0–100% linear gradient of solvent A (double-distilled water, 0.1% formic acid) to B (acetonitrile, 0.1% formic acid) was employed over 20 min, returning to 100% A in 2 min and a 6-min post-time solvent A hold. The UHPLC system was coupled online with a mass spectrometer Q-Exactive (Thermo Fisher Scientific, Waltham, MA, USA) scanning in full MS mode (2 μscans) at 70,000 resolution in the 67 to 1000 *m/z* range, target of 1 × 10^6^ ions and a maximum ion injection time (IT) of 35 ms. Source ionization parameters were: spray voltage, 3.8 kV; capillary temperature, 300 °C; sheath gas, 40; auxiliary gas, 25; S-Lens level, 45. Calibration was performed before each analysis against positive ion mode calibration mixes (Piercenet, Thermo Fisher Scientific, Waltham, MA, USA) to ensure sub ppm error of the intact mass.

### 2.6. Metabolomic Data Processing and Statistical Analysis

Raw files of replicates were exported and converted into mzXML format through MassMatrix (Cleveland, OH), then processed by MAVEN.52 (available at http://genomics-pubs.princeton.edu/mzroll/) [44]. Spectrograms were analyzed for peak alignment, matching and comparison of parent and fragment ions, and for tentative metabolite identification (within a 2 ppm mass-deviation range between observed and expected results against the imported KEGG database). Results were graphed with Graphpad Prism 5.01 (Graphpad Software Inc, San Diego, CA, USA). Verification of accepted metabolites was conducted manually using HMDB, KEGG, and PubChem DBs.

### 2.7. Plant Fitness Parameters

Leaf areas were measured by using a ruler. The flowering time was calculated as the time of appearance of the first flower after the treatment. Leaf dry weight was determined on a microbalance with a 0.001 g resolution when the leaves had fully dried in a 60 °C stove. To determine seed production, plants were watered every other day until they stopped producing new flowers. Inflorescences were harvested when all plants had finished flowering and the seeds were weighed on a microbalance with a 0.0001 g resolution. 

### 2.8. ROS Detection in Arabidopsis Leaves 

ROS detection was performed as previously described [45,46]. Briefly, ROS production was revealed by the specific probe 2′,7′-dichlorofluorescein diacetate (DCFH_2_-DA; Sigma Aldrich, St. Louis, MO, USA), which is rapidly oxidized to highly fluorescent dichlorofluorescein (DCF) in the presence of ROS. Five-week-old Col-8 and *glyI4* leaves were harvested. Two leaves from each of six plants were collected. One leaf from each plant was incubated at room temperature in a solution of 20 mM DCFH_2_-DA in 10 mM Tris-HCl (pH 7.4) for 45 min in the dark. As a negative technical control, the remaining half was incubated in 10 mM Tris-HCl (pH 7.4) only, under the same conditions. After staining, the samples were washed three times in fresh buffer for 10 min to remove the excess of fluorophore and mounted on glass slides. Fluorescence was then observed under a LSM 710 confocal microscope (Carl Zeiss Microscopy GmbH, Jena, Germany) with Plan Neofluar 20/1.30 objective. Two laser excitations lines were used (i.e., 488 nm for probe detection and 561 nm for chlorophyll auto-fluorescence). Data were processed using Image J software (http://rsbweb.nih.gov/ij/). 

### 2.9. Stomata Staining

Leaves from five-week-old Col-8 and *glyI4* plants were collected. Epidermal peels were obtained from intact leaves by scraping on the abaxial sides using precision tweezers. The isolated adaxial epidermis was stained with 0.1% (*w/v*) acridine orange in water (Biotium, Fremont, CA, USA) for 15 min; then were washed twice in distilled water for 10 min, mounted on glass slides. Fluorescence was observed under a LSM 710 confocal microscope (Carl Zeiss Microscopy GmbH, Jena, Germany) with Plan Neofluar 20/1.30 or 63/1.30 objective. Two laser excitations lines were used (i.e., 488 for AO staining and 561 nm for chlorophyll autofluorescence). Data were processed using Image J software (http://rsbweb.nih.gov/ij/). 

### 2.10. Resistance Assays

Infections with the fungus *Plectosphaerella cucumerina* BMM or the bacterium *Pseudomonas syringae pv. tomato* (Pst) DC3000 were performed on five-week-old Col-8 and *glyI4* plants. *P. cucumerina* conidia were collected from fungal cultures grown on half strength potato dextrose agar (PDA), at 20 °C, and suspended in 0.5× potato dextrose broth (PDB) (Sigma, Steinheim, Germany) at a concentration of 5 × 10^6^ conidia/mL. Infections were carried out by applying a single 6-µL drop of conidial suspension on three to five leaves per plant, on the adaxial leaf surface. Plants were incubated under high relative humidity (RH) conditions (˃80%) in closed boxes for seven days, after which the diameter of the necrotic lesions were determined. Pst DC3000 was grown overnight in King’s medium B (12 mM MgSO_4_ × 7H_2_O, 8 mM K_2_HPO_4_, 81 mM peptone, and 100 mM glycerol) containing 60 µM of rifampicin. Bacterial suspension was centrifuged at low speed and washed twice in 10 mM MgCl_2_. Infections were carried out by dipping plant leaves for 4 s in a bacterial suspension containing 10^6^ colony-forming units (CFU)/mL in 10 mM MgCl_2_ and 0.01 % *v*/*v* of the surfactant Silwet L-77 (Van Meeuwen Chemicals BV, Weesp, the Netherlands). Infected plants were incubated under high RH (˃80%) in closed boxes for the first 6 h. After 2 h (time zero) and 72 h the whole aerial part of each plant was harvested, weighed, washed twice, and ground in 10 mM MgCl_2_ in order to extract bacterial cells. Serial dilutions were plated on King’s B agar containing 50 µg/mL of rifampicin in order to count CFU and determine the bacterial load of each plant (expressed as Log CFU/mg). 

### 2.11. GLYI4 Subcellular Localization 

The *GLYI4* coding sequence was subcloned into the pUC57 vector (pUC57::GLYI4, purchased from Synbio Technologies, Monmouth Junction, NJ, USA). GLYI4 coding sequence was excised from pUC57::GLYI4 vector by digestion with PinAI enzyme and ligated into the same sites of the pUC-35S::EYFP vector, upstream and in frame to EYFP sequence under the control of 35S promoter of cauliflower mosaic virus. The vector (pUC-35S::GLYI4-EYFP) was fully sequenced to confirm the correct frame of the insert. 

Cellular localization of GLYI4 was investigated by protoplast transient expression approach followed by confocal microscope observation of the transformed fluorescent protoplasts. Protoplasts were isolated from five-week-old *glyI4* mutant and transformed with the vector (pUC-35S::GLYI4-EYFP) using the polyethylene glycol (PEG)-calcium transfection protocol described [47]. PEG-calcium transfection was performed with protoplasts at a density of 1 × 10^6^/mL. Two different sets of protoplasts were independently transformed with the vector. One set was treated with 100 µM MeJA prior transformation and the other one was treated with water. Protoplasts were harvested 20 h after transformation and were analyzed for chimeric protein localization using a Zeiss LSM 710 confocal microscope (Carl Zeiss Microscopy GmbH, Germany) with Plan Neofluar × 40/1.30 objective. Two laser excitations lines were used (i.e., 514 nm for EYFP, and 563 nm for chloroplast autofluorescence). Data were processed using Image J software (http://rsbweb.nih.gov/ij/).

### 2.12. Statistical Analyses

Expression of *PDF1.2* and *GLYI4* genes have been compared between treatments and between genotypes using two-way analysis of variance (ANOVA) and Tukey’s multiple-comparison posttest. Differences between data were significant at a *p* value of < 0.0001. *Pst DC3000* bacterial proliferation has been compared between time points and between genotypes using two-way analysis of variance and Tukey’s multiple-comparison posttest. Differences between data were significant at a *p* value of <0.0001. Student’s t-test for normally distributed data was performed when two groups were considered. Differences were considered when * *p* < 0.05; ** *p* < 0.005; *** *p* < 0.005. Statistical analyses were performed with GraphPad Prism 7.0 (GraphPad Software Inc., San Diego, CA, USA).

## 3. Results

### 3.1. GLYI4 Acts in the MG Scavenging Pathway

**Figure 1:**
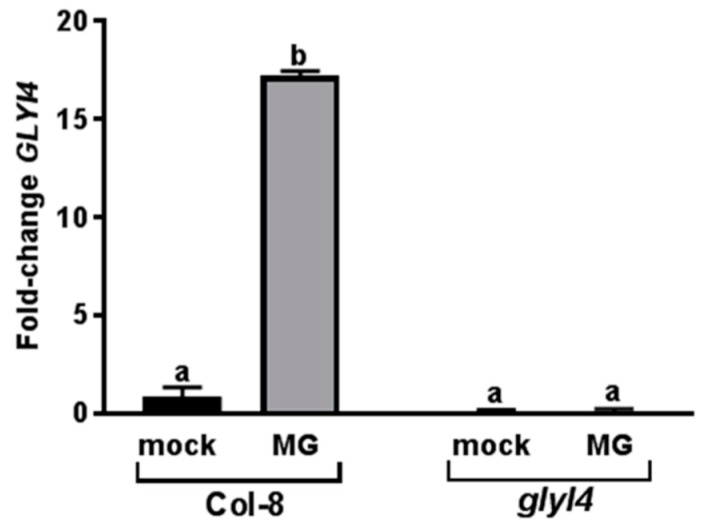
Glyoxalase (*GLYI4)* gene expression analysis in Arabidopsis Col-8 and *glyI4* mutant after methylglyoxal (MG) treatment. qRT-PCR analysis of *GLYI4* in Col-8 and *glyI4* leaves treated with MG and a mock solution. Fold change is relative to the expression in mock-treated plants and normalized to the reference gene *PP2AA3*. *GLYI4* gene expression analyses were performed 24 h after MG treatment of five-week-old plants. Shown data are means of three biological replicates. Error bars represent SEM. Different letters indicate significant statistical differences (Two-ways ANOVA, Tukey’s post-hoc, *p* < 0.0001).

To investigate whether GLYI4 plays a role in the MG detoxification pathway, we first tested the effect of MG on *GLYI4* expression in wild-type Arabidopsis accession Col-8 and in *glyI4* mutant. *GLYI4* expression was monitored in 5-week-old plants, 24 h after exogenous application of MG, and compared with the *GLYI4* expression level in mock-treated. In Col-8, *GLYI4* transcript was significantly induced after MG treatment showing 18-fold induction compared to mock, while in *glyI4* mutant was not detectable (Figure 1). 

**Figure 2:**
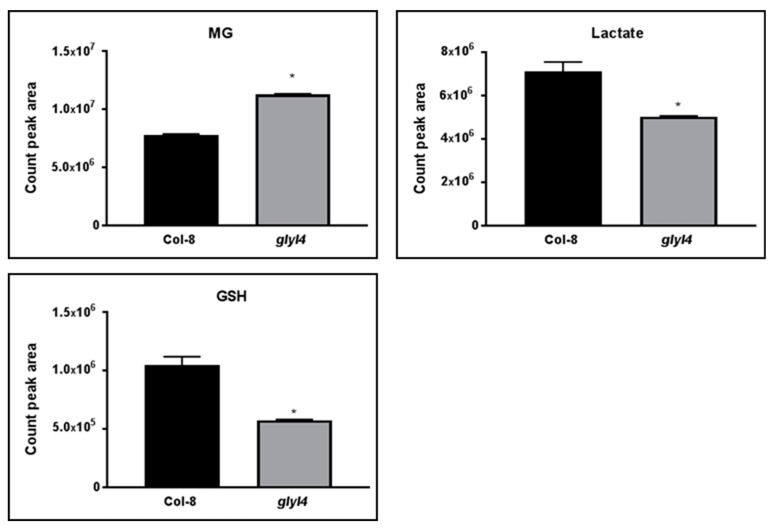
Analysis of MG-scavenging pathway metabolites in Arabidopsis Col-8 and *glyI4* mutant. MG, lactate, and glutathione (GSH) amounts were analyzed in Col-8 and *glyI4* leaves. Shown data are means of three biological replicates. Error bars represent SEM. Asterisk indicates the statistically significant difference between Col-8 and *glyI4* plants (*t*-test; * *p* < 0.05).

In order to confirm the importance of GLYI4 in MG scavenging, MG and two other key compounds of the scavenging pathway (lactate and -GSH-) were analyzed in the T-DNA insertion mutant *glyI4* and in Col-8, by LC-MS/MS. MG is detoxified via the glyoxalase system composed of glyoxalase I and glyoxalase II, which catalyze the detoxification of MG to D-lactate using GSH as a cofactor. At the end of the reaction, GSH is recycled because the availability of GSH is an important factor for detoxifying MG via the glyoxalase system [48]. We found that the concentration of MG detected in *glyI4* mutant was higher than in Col-8. By contrast, the levels of lactate and GSH were lower in *glyI4*, compared to that in Col-8 (Figure 2). 

**Figure 3:**
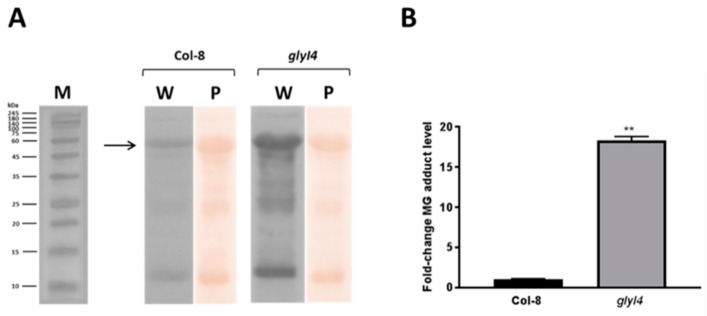
MG-protein adducts level in Arabidopsis Col-8 and *glyI4* leaves. (**A**) Western blot performed with anti-methylglyoxal monoclonal antibody using Col-8 and *glyI4* total protein extracts (40 g) from five-week-old plant leaves (W). Ponceau S staining (P) was used to normalize protein loading. Protein molecular weight markers are also shown (M). (**B**) Densitometric analysis of the most intense MG protein-adduct band, as indicated by the arrow in A. Fold-change is relative to the expression in Col-8 plants and normalized to the Ponceau stained band. Shown data are means of three biological replicates. Error bars represent SEM. Asterisks indicate the statistically significant difference between Col-8 and *glyI4* (t-test; ** *p* < 0.005).

MG accumulation can be detrimental by reacting with several macromolecules causing their inactivation or degradation. In fact, it has been reported that MG forms adducts with arginine, lysine and cysteine residues of proteins, generating advanced glycation end-products (AGEs) [10,23]. AGEs adversely affect the whole organism, causing oxidative damage to key cellular components [49,50,51]. In order to assess the accumulation of MG-protein adducts in *glyI4* mutant, Western blot analyses with antibody detecting MG-adducts were carried out (Figure 3). This antibody specifically recognizes methylglyoxal 5-hydro-5-methylimidazolones (MG-H1), formed with the amino groups of the arginine side chain.

As shown in Figure 3A, there was a significant net increase in MG-protein adducts in *glyI4* mutant compared to Col-8. Moreover, the densitometric analysis carried out on the most intense protein band detected on the membrane showed an 18-fold increase of adduct formation in *glyI4*, compared to Col-8 (Figure 3B). 

### 3.2. GLYI4 Impacts Fitness and Physiology of Arabidopsis Plants

**Figure 4:**
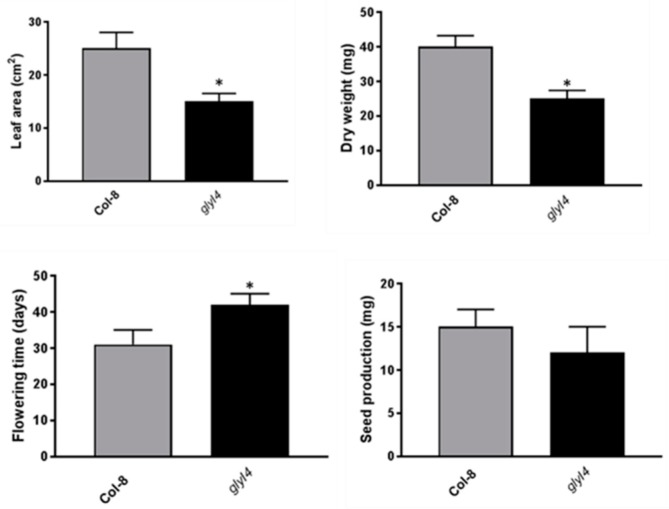
Fitness parameters of Arabidopsis Col-8 and *glyI4*. Leaf area (cm^2^), dry weight (mg), flowering time (days), and seed production (mg), per plant, were analyzed in Col-8 and *glyI4* plants. Error bars represent SEM. Asterisk indicates a statistically significant difference between *glyI4* and Col-8 (t-test; * *p* < 0.05, *n* = 15). The experiments have been repeated three times with similar results.

To investigate whether MG accumulation impacted the fitness of the plants, leaf area, dry weight, flowering time, and seed production were measured in five-week-old *glyI4* and Col-8 plants at selected timing. As shown in Figure 4, *glyI4* phenotype was characterized by a significantly reduced leaf area and dry weight, as well as prolonged flowering time compared to Col-8. 

Moreover, we observed a moderate, but not statistically significant, reduction in seed production. Thus, MG accumulation had a negative effect on the analyzed fitness parameters indicating a moderate stress phenotype of *glyI4* plants. 

In the presence of an excess of MG, plants have been reported to be subjected to a strong oxidative stress, due to an enhanced reactive oxygen species production [48]. In order to demonstrate MG-related ROS increase in the *glyI4* mutant with respect to Col-8, five-week-old Arabidopsis leaves were incubated with 2′,7′-dichlorofluorescein diacetate (2′,7′-DCFH_2_-DA).This compound and its derivatives are largely used as ROS-sensitive dyes [52].This compound diffuses through the plasma membrane into the cytoplasm where is deacetylated by intracellular esterase and then oxidized by ROS producing the green fluorescent dye 2′,7′-DCF. Originally, oxidation of DCFH_2_ to DCF was thought to be specific for H_2_O_2_, but recent evidence has shown that other ROS such as hydroxyl radical, hydroperoxides, and peroxynitrite can also oxidize DCFH_2_, although with greatly reduced sensitivity as compared with that of H_2_O_2_ [53]. 

**Figure 5:**
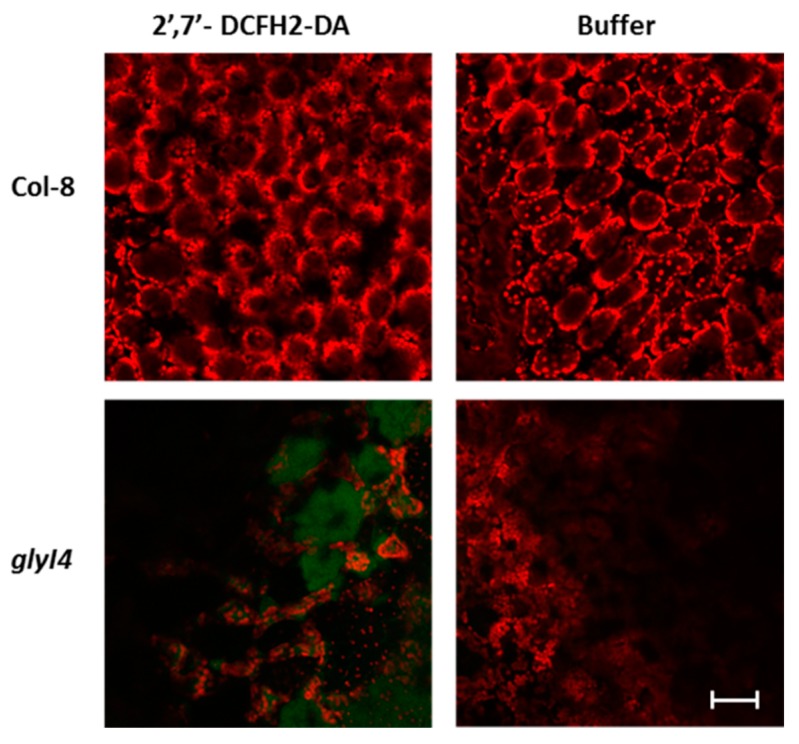
Detection of ROS in Arabidopsis Col-8 and *glyI4* leaves. Detection on Col-8 and *glyI4* leaves was carried out by using 2′,7′-DCFH_2_-DA or buffer (negative technical control). Fluorescence was observed under an LSM 710 confocal microscope with Plan Neofluar 20/1.30 objective. Two laser excitations lines were used [i.e., 488 for probe detection (green) and 561 nm for chlorophyll autofluorescence (red)]. Bar corresponds to 50 μm.

Col-8 plants treated with buffer showed the red fluorescence due to chlorophyll only, while *glyI4* mutants showed very strong green fluorescence due to 2′,7′-DCF highlighting the presence of high levels of ROS (Figure 5).

**Figure 6:**
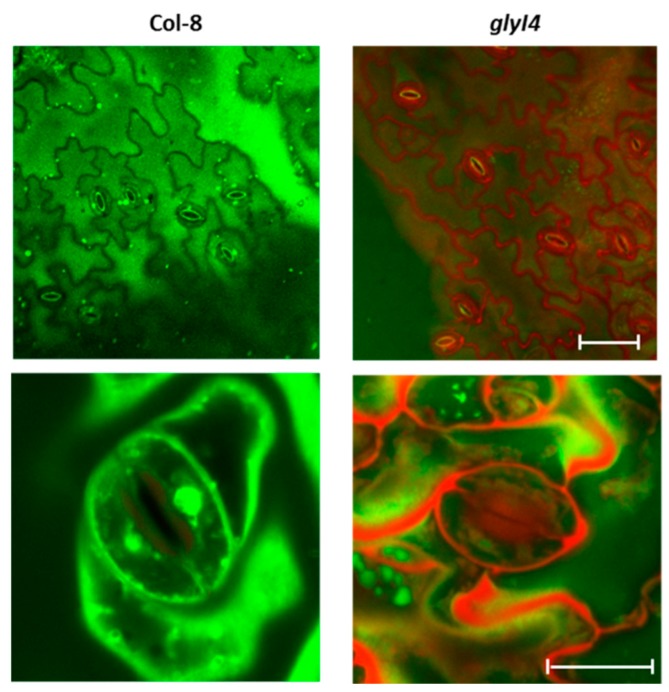
Confocal microscope representative images of stomatal guard cells of Arabidopsis Col-8 and *glyI4* leaves. Abaxial peeled epidermis of Col-8 and *glyI4* leaves was stained with acridine orange (AO) and observed by the confocal microscope. AO is colored in green in not acidified cell compartments while AO red color indicates an acidification of cellular compartments. Magnification of guards cells is also shown (lower panels). Fluorescence images were acquired using a Zeiss LSM 710 confocal microscope with Plan Neofluar 20/1.30 objective (upper panel) and Plan Neofluar 63/1.30 objective (lower panel). Fluorescence emissions of AO in the red and green channels (615 to 660 nm and 530 to 540 nm, respectively) after excitation with a 488-nm laser were obtained. Bar corresponds to 100 μm (upper panels) or 10 μm (lower panels).

It has been reported that MG can induce stomatal closure by modulating ROS production and cytosolic free Ca^2+^ concentration increase in the Arabidopsis leaf guard cells [48]. Moreover, MG can cross both prokaryotic and eukaryotic membranes leading to increased acidification in the cellular compartments related to stomatal closure [48,54,55,56,57,58]. Thus, we addressed the question whether MG accumulation affected stomatal behavior in our model system. The fluorochrome acridine orange (AO) has been widely exploited to stain stomata in diverse plant species [58]. AO is a cell permeable pH-sensitive fluorescent dye and has a maximum emission at 540 nm (green light) in its neutral form. However, when AO enters acidic compartments, it becomes protonated remaining sequestered in these compartments, shifting its maximum emission to 660 nm (red light). When the abaxial epidermis of *glyI4* and Col-8 leaves was peeled off and stained with AO, red fluorescence was observed only in stomata of the *glyI4* mutant at the level of outer cell layers as well as in the cell compartments close to stomatal aperture, indicating their acidification (Figure 6).

### 3.3. GLYI4 Affects JA-Dependent Defense Responses

**Figure 7:**
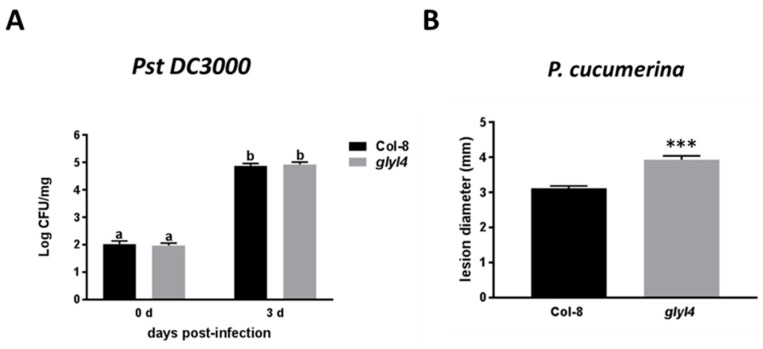
Bacterial proliferation and disease rate in Arabidopsis Col-8 and *glyI4* plants infected with *Pst DC3000* and *P. cucumerina,* respectively. (**A**) Plants were challenged with *Pst DC3000 by* dipping the aerial part in a bacterial suspension (10^6^ colony-forming units (CFU)/mL). The values reported represent means of the log of the proliferation values, recorded at zero and three days post inoculation. Data represent the average from six infected plants. The experiment was repeated two more times with similar results. Error bars represent SEM. Different letters mean significant statistical differences (Two-ways ANOVA, Tukey’s post-hoc, *p* < 0.0001). (**B**) Plants were infected with *P. cucumerina* by inoculating leaves with 6 L-drop of a suspension containing 5 × 10^6^ conidia/mL. Disease symptoms were recorded at seven days post inoculation by measuring the diameter of the necrotic lesions (mm). The figure shows a representative experiment from three independent repetitions. Data represent the average from 45 lesions produced on 12 plants per genotype. Error bars represent SEM. Asterisk indicates significant statistical differences compared with Col-8 (t-test, *** *p* < 0.0001).

GLYI4 was demonstrated to play a role in SA-mediated suppression of JA signaling pathway and in the defense response against fungal pathogen triggering both hormones [38]. Moving further, we asked a question whether GLYI4 could influence the plant defense reaction against pathogens that trigger SA or JA signaling pathways. For this purpose, *glyI4* mutant plants were tested for their level of resistance against *Pseudomonas syringae* pv. *tomato* strain DC3000 (*Pst* DC3000) and *Plectosphaerella cucumerina* (*P. cucumerina*). *Pst* DC3000 is known to activate SA signaling [59], while JA is the main hormone mediating resistance against *P. cucumerina* [60,61]. When five-week-old *glyI4* and Col-8 plants were infected with *Pst DC3000* and colony forming units were counted three days later there were no significant differences between the mutant and wild-type plants (Figure 7A).

**Figure 8:**
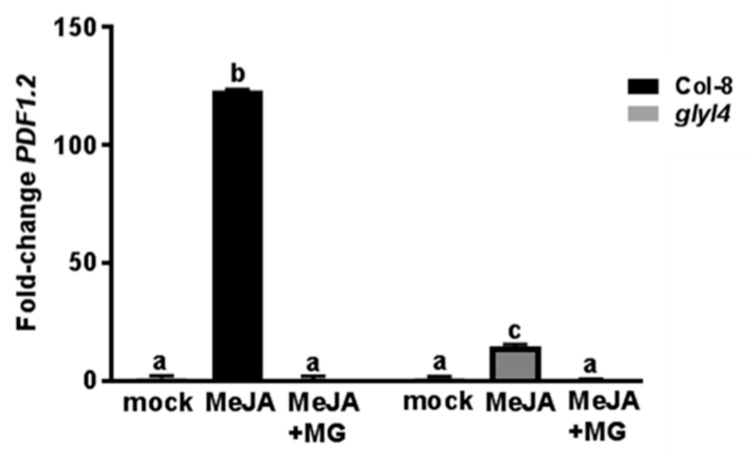
*PDF1.2* gene expression analysis in *Arabidopsis* Col-8 and *glyI4* mutant after methyl jasmonate (MeJA) and MeJA+MG treatment. qRT-PCR analysis of *PDF1.2* expression in Col-8 and *glyI4* leaves treated with a mock solution, MeJA or MeJA+MG. Fold change is relative to the expression in mock-treated plants and normalized to the reference gene *PP2AA3*. *PDF1.2* gene expression analyses were performed 24 h after each treatment using five-week-old plants. Shown data are means of three biological replicates. Error bars represent SEM. Different letters indicate statistically significant difference between treatments (two-way ANOVA; Tukey’s test *p* < 0.0001).

This result strengthens previous findings that indicate no significant alteration of the SA signaling pathway in *glyI4* mutants, compared to Col-8 [38]. Moreover, when five-week-old *glyI4* and Col-8 plants were inoculated with *P. cucumerina* spores, and disease symptoms were scored seven days later, *glyI4* mutant plants developed significantly more severe disease symptoms than Col-8 (Figure 7B), thus suggesting that GLYI4 loss-of-function negatively affects JA pathway responses. To confirm this observation, *PDF1.2* expression was evaluated as it represents the principal marker of this pathway. As expected, in Col-8 plants this marker gene was highly induced upon MeJA treatment, but interestingly its expression was significantly suppressed by MG, indicating an antagonistic role of this compound on JA-mediated defenses. Noteworthy, in *glyI4* mutant MeJA-induced *PDF1.2* expression was significantly reduced compared to that in Col-8 (Figure 8). 

**Figure 9:**
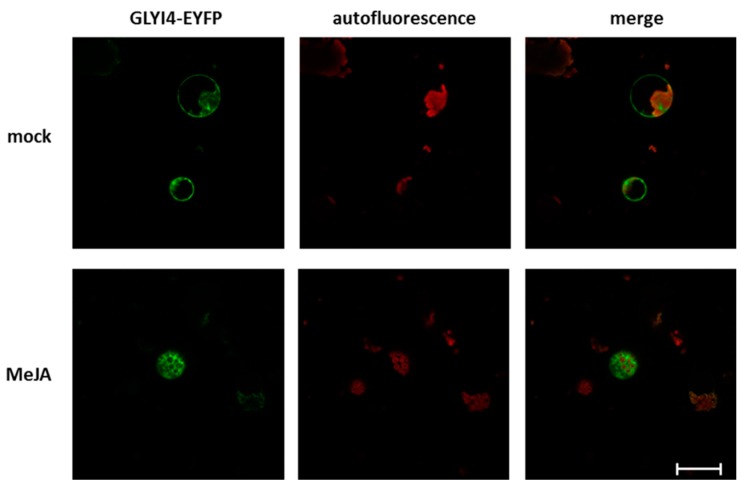
GLYI4 localization in Arabidopsis protoplasts. *glyI4* leaf protoplasts were MeJA- and mock-treated and then transformed with pUC-35S::GLYI4- enhanced yellow fluorescent protein (EYFP). Fluorescence images were acquired using a Zeiss LSM 710 confocal microscope with Plan Neofluar 40/1.30 objective. Two laser excitation wavelengths were used (i.e., λexc = 514 nm for EYFP (green), and λexc = 561 nm for chloroplast autofluorescence (red)). The merged images are also presented. Bars correspond to 50 μm.

Finally, to gain more insight into the molecular function of GLYI4, we determined the GLYI4 subcellular localization. *GLYI4* cDNA was cloned in a plasmid upstream of the enhanced yellow fluorescent protein (EYFP) coding sequence. The resulting chimeric protein GLYI4-EYFP was transiently expressed into *glyI4* Arabidopsis mesophyll protoplasts, MeJA-treated for 6 h prior to the transformation and in mock-treated. Figure 9 shows that in mock-treated protoplasts GLYI4 was mainly localized to the plasma membrane, where it is likely to play a role in the MG detoxification pathway. Interestingly, in MeJA-treated protoplasts the protein accumulated in the cytoplasm.

## 4. Discussion

Methylglyoxal production is an unavoidable consequence of metabolism, even under normal physiological conditions in any organisms. Nevertheless, excessive MG accumulation in plant cells can have detrimental effects on plant physiology, leading to disruption of several cellular functions [8]. The glyoxalase enzymes belonging to GLY I and GLY II families act coordinately to detoxify MG [48]. Recently, by GWA study we identified the glyoxalase GLYI4 as a positive regulator of the SA-mediated antagonism on JA pathway, with potential effect in the regulation of pathogen resistance [38]. However, GLYI4 has been poorly characterized, so it is worthy to gain deeper into the involvement of GLYI4 in both MG scavenging and in JA signaling, to unravel a possible role of this enzyme as hub in both pathways. 

In this work, we investigated the impact of GLYI4 on MG scavenging. *GLYI4* gene was highly expressed in Arabidopsis Col-8 after exogenous treatment with MG, suggesting that the toxic compound regulates its expression (Figure 1). This is consistent with the observation that GLYI genes from *O. sativa* were up-regulated after MG stress [37]. Moreover, we found that MG level is higher in *glyI4* knock-down mutant than in Col-8 plants, while the levels of its detoxification products, i.e., lactate and GSH, were lower compared to that in Col-8 (Figure 2). Thus, our results underline that a loss-of-function of GLYI4 leads not only to MG accumulation, but also to a general alteration in the amounts of the final products of the scavenging pathway. Accumulation of MG has been also reported in yeast mutants impaired in MG detoxification enzymes [11]. Furthermore, MG-mediated GSH depletion has been already observed in human cells [62]. The accumulation of MG is often called dicarbonyl stress, which has been implicated as a cause of tissue damage and aging [63]. The dicarbonyl group within MG can readily react with the amine groups of proteins, lipids and nucleic acids, forming advanced glycation end products, which can cause oxidative damage to key cellular components [49,50]. In particular, MG preferably reacts with arginine, lysine and cysteine residues of proteins to form AGEs. The result obtained by Western blotting confirmed that *glyI4* mutant shows higher accumulation of MG-protein adducts, compared to the control (Figure 3). Taken together, these results indicate that although there are 22 GLYI protein members in Arabidopsis that could potentially work redundantly, it seems that the lacking of *GLYI4* is crucial for MG accumulation. 

As widely reported, high levels of intracellular MG strongly impair plant growth and development [48,64,65]. Accordingly, we found a reduced plant fitness of *glyI4* mutants, compared to Col-8. Plant fitness can be measured by morphometric parameters such as growth, flowering time and seed set, suggesting a typical stress phenotype in *glyI4* mutant characterized by lower vegetative development, early flowering time, and lower seed production (Figure 4). This suggests that *GLYI4* could be a crucial component for a successful plant development and fitness. Under stress, MG increases ROS formation in plant cells which also play a key role in plant physiology and in stress responses [66]. One of the best investigated ROS is hydrogen peroxide (H_2_O_2_), which accumulates in response to stress and it is responsible for several phenomena, including stomatal closure and cell death [67]. It has been found that MG-induced stomatal closure accompanied by production of ROS and cellular compartment acidification [7,54,55,56,57,58]. In our study, we observed that the *glyI4* mutant showed both increased ROS formation and stomatal closure (Figure 5; Figure 6, respectively), likely due to MG accumulation. Stomatal closure is a unique adaptive phenomenon in plants to withstand extreme stress conditions [68]. MG-induced stomatal closure may contribute to plant adaptation during stress by preventing water loss through transpiration and representing also an effective barrier against pathogens [7]. 

On the other hands, plant responses to biotic stresses are very complex and involve numerous signal molecules, often communicating with each other. Based on this, it is possible to hypothesize that MG cooperates with other compounds in mediating defense responses or susceptibility against biotic threats. In order to characterize the resistance of *glyI4* mutants against biotic cues, we used the bacterial pathogen *Pst DC3000* and the fungal necrotrophic pathogen *P. cucumerina*. Resistance against *Pst DC3000* is mainly orchestrated by SA [59], while JA pathway mainly contributes to the host resistance against *P. cucumerina* [60,61]. Interestingly, *glyI4* mutant showed significant increased susceptibility to *P. cucumerina*, compared to Col-8 while no difference in resistance against *Pst DC3000* was found (Figure 7). These results strengthen previous findings that demonstrated a significant impairment of the JA-dependent defense responses in *glyI4* mutant compared to Col-8, while SA-dependent responses were not affected [38]. In order to highlight the involvement of MG on JA defense pathway, the expression of *PDF1.2* was evaluated in Col-8 plants after MeJA alone or in combination with MG. MG induced a strong inhibition of the MeJA-induced expression of *PDF1.2*, highlighting an antagonistic interaction on the JA pathway (Figure 8). In this perspective, the interaction between MG and hormone pathways could represent a barrier that prevents defense against necrotrophic pathogens. 

Finally, to help unravel the function of GLYI4, we investigated its subcellular localization in protoplasts of *glyI4* mutants. Interestingly, we found that GLYI4 accumulated at the plasma membrane (Figure 9), suggesting that MG detoxification and signaling could also take place in this compartment, by recruiting the first enzyme of the MG scavenging pathway. Previous studies demonstrated MG accumulation and activity at the level of the plasma membrane, where the compound can bring about lipid peroxidation [69]; in fact, MG rapidly reacts with membrane phospholipids, producing AGEs leading to membrane dismantling [70]. Interestingly, there is evidence that AGEs interact with plasma membrane receptors called receptor for advanced glycation end products (RAGEs), leading either to their endocytosis and degradation or to immune responses [71,72]. A striking feature of RAGE signaling is its multi-ligand capacity. Interestingly, they are classified as receptors that respond to damage associated molecular patterns (DAMPs) and which are able to activate MAPK cascades, inducing inflammatory cytokines [72]. Most of the RAGE ligands are involved in inflammation and cell migration processes [73], therefore it is not surprising that RAGEs are enrolled in defense mechanisms. Interestingly, after MeJA stimulus GLYI4 translocated to the cytoplasm (Figure 9), where MeJA signaling usually takes place [74,75]. The signal for GLYI4 re-localization could be either an accumulation of MG due to MeJA or an involvement of GLYI4 in MeJA signaling pathway. This translocation could be possible due to post-translational modifications (PTMs)of GLYI4. In fact, members of Arabidopsis glyoxalase I family are subjected to phosphorylation [76], which plays important roles in regulation of cell signaling and growth. Moreover, PTMs can lead to modification of enzyme activity, protein structure, and protein-protein interaction that can lead to cellular re-localization and new biological function [76]. In conclusion, our results support the emerging novel cross-communication between MG and MeJA. Accumulation of MG in *glyI4* mutant clearly impacts the JA signaling pathway in Arabidopsis. Furthermore, we demonstrated that MG levels and JA pathway are closely associated with biotic stress responses. In the future, in-depth understanding on how MG and JA pathway cross-communicate will likely reveal other intriguing regulatory roles for GLYI4 as a potential hub.
